# MiR‐34b/c‐5p and the neurokinin‐1 receptor regulate breast cancer cell proliferation and apoptosis

**DOI:** 10.1111/cpr.12527

**Published:** 2018-10-17

**Authors:** Lufang Zhang, Lushan Wang, Dong Dong, Zhiyong Wang, Wei Ji, Man Yu, Fei Zhang, Ruifang Niu, Yunli Zhou

**Affiliations:** ^1^ Department of Clinical Laboratory, Tianjin Medical University Cancer Institute and Hospital, National Clinical Research Center for Cancer, Tianjin’s Clinical Research Center for Cancer, Tianjin Key Laboratory of Cancer Prevention and Therapy, Key Laboratory of Breast Cancer Prevention and Therapy of Educational Ministry Tianjin Medical University Tianjin China; ^2^ Department of Clinical Laboratory Aviation General Hospital Beijing China; ^3^ Public Laboratory, Tianjin Medical University Cancer Institute and Hospital, National Clinical Research Center for Cancer, Tianjin’s Clinical Research Center for Cancer, Tianjin Key Laboratory of Cancer Prevention and Therapy, Key Laboratory of Breast Cancer Prevention and Therapy of Educational Ministry Tianjin Medical University Tianjin China; ^4^ Department of Laboratory Medicine and Pathobiology University of Toronto Toronto ON Canada

**Keywords:** aprepitant, cell apoptosis, cell proliferation, miR‐34, neurokinin‐1 receptor, Substance P

## Abstract

**Objectives:**

MiR‐34 is a tumour suppressor in breast cancer. Neurokinin‐1 receptor (NK1R), which is the predicted target of the miR‐34 family, is overexpressed in many cancers. This study investigated the correlation and clinical significance of miR‐34 and NK1R in breast cancer.

**Materials and Methods:**

Western blotting, quantitative reverse transcription‐PCR (qRT‐PCR) and luciferase assays were conducted to analyse the regulation of NK1R by miR‐34 in MDA‐MB‐231, MCF‐7, T47D, SK‐BR‐3 and HEK‐293 T cells. MiR‐34b/c‐5p, full‐length NK1R (NK1R‐FL) and truncated NK1R (NK1R‐Tr) expression in fifty patients were quantified by qRT‐PCR and correlated with their clinicopathological parameters. CCK‐8 assays, colony formation assays and flow cytometry were used to measure cell proliferation and apoptosis in MDA‐MB‐231 and MCF‐7 cells transfected with miR‐34b/c‐5p or NK1R‐siRNA and before treatment with or without Substance P (SP), an endogenous peptide agonists of NK1R. The effect of NK1R antagonist aprepitant was also investigated. In vivo xenograft models were used to further verify the regulation of NK1R by miR‐34b/c‐5p.

**Results:**

Expression levels of miR‐34b/c‐5p and NK1R‐Tr, but not NK1R‐FL, were associated with enhanced malignant potential, such as tumour stage and Ki67 expression. The overexpression of miR‐34b/c‐5p or NK1R silencing potently suppressed cell proliferation and induced G2/M phase arrest and the apoptosis of MDA‐MB‐231 and MCF‐7 cells. The NK1R antagonist aprepitant had similar effects. In vivo studies confirmed that miR‐34b/c‐5p overexpression or NK1R silencing reduced the tumorigenicity of breast cancer. In addition, SP rescued the effects of miR‐34b/c‐5p overexpression or NK1R silencing on cell proliferation and apoptosis in vitro and in vivo assays.

**Conclusions:**

MiR‐34b/c‐5p and NK1R contribute to breast cancer cell proliferation and apoptosis and are potential targets for breast cancer therapeutics.

## 1 INTRODUCTION

Breast cancer is the most common cancer among women. Each year, more than 200 000 new cases are diagnosed and more than 40 000 women die from breast cancer.^1^ However, rapid disease progression remains a major obstacle in the successful treatment of breast cancer and it is still important to investigate the molecular mechanisms that suppress cell proliferation and govern apoptosis.

MicroRNAs (miRNAs) are a class of endogenous, small, noncoding RNAs. They modulate cell behaviours, proliferation, differentiation and apoptosis by simultaneously targeting multiple genes.^2^, ^3^, ^4^, ^5^ In breast cancer, the miR‐34 family is often lost or poorly expressed and is a potential tumour suppressor gene.^4^, ^6^ MiR‐34 may inhibit breast cancer cell proliferation by suppressing c‐Myc and the phosphatase activities of ERK and AKT.^7^, ^8^ Moreover, miR‐34 induces cell cycle arrest and apoptosis in breast cancer.^4^, ^9^ These findings strongly suggest that miR‐34, as a tumour suppressor, may be a new therapeutic target and intervention strategy against breast cancer.

G protein‐coupled receptors (GPCR) are overexpressed in various cancers and contribute to cell growth when they are activated by circulating or locally produced ligands; they are also used as a tumour diagnosis signal,^10^, ^11^, ^12^ although the underlying molecular mechanisms remain poorly understood. One such GPCR is neurokinin‐1 receptor (NK1R)^13^, ^14^ and its specific endogenous peptide agonist is neuropeptide Substance P (SP).^15^ Evidence indicates that the biological action of SP is primarily mediated by its binding to NK1R.^16^ The SP‐NK1R signalling pathway is linked to the pathophysiological processes of pain transmission,^7^ chemotherapy‐induced vomiting^17^ and inflammation.^18^ There is a close correlation between SP‐NK1R and tumour development.^15^, ^19^, ^20^ NK1R expression is elevated in glioblastoma^15^ and pancreatic,^21^ breast^22^, ^23^ and gastric cancers.^24^ SP promotes cell proliferation and increases collagen I expression.^25^ NK1R agonists promote glioblastoma cell proliferation and migration, whereas the NK1R antagonists L732138 and SPA induce cell apoptosis.^15^ Moreover, another NK1R antagonist, aprepitant, induces robust inhibition of tumour growth and apoptosis in a wide variety of cancers.^26^, ^27^, ^28^, ^29^ NK1R has two structural isoforms, the full‐length receptor (NK1R‐FL) and the truncated receptor (NK1R‐Tr). In addition to their structural differences, the two isoforms also exert different biological functions. We and others have demonstrated that NK1R‐Tr expression is upregulated in cancer.^22^, ^23^ Our previous study shows that NK1R‐Tr is strongly overexpressed in breast cancer. Furthermore, NK1R is a putative target of miR‐34, as determined by analyses using several bioinformatics databases such as TargetScan, miRbase and RNAhybrid. The aim of our study was to investigate the biological roles of miR‐34 and NK1R in breast cancer. We also explored the potential regulation of NK1R by miR‐34 and their potential as therapeutic targets for breast cancer patients.

In this study, we found that the expression of NK1R‐Tr was markedly upregulated and that the levels of miR‐34b/c‐5p and NK1R‐FL were downregulated in human breast cancer cell lines and tumour tissues. However, miR‐34b/c‐5p and NK1R‐Tr, but not NK1R‐FL, were significantly associated with the clinical features of breast cancer. However, the inhibition of cell proliferation by miR‐34b/c‐5p overexpression was primarily consistent with the silencing of NK1R‐Tr, but not NK1R‐FL, in breast cancer and miR‐34b/c‐5p overexpression and NK1R‐Tr silencing both induced cell cycle G2/M arrest and apoptosis; furthermore, SP only partly rescued this effect. Aprepitant, an antagonist of NK1R, also inhibits cell proliferation and induces cell cycle G2/M phase arrest and apoptosis. Finally, animal experiments further confirmed the tumour inhibition effects of miR‐34b/c‐5p and NK1R silencing. Together, our data indicate that miR‐34b‐5p, miR‐34c‐5p and NK1R‐Tr play important roles in the initiation and proliferation of human breast cancer, and they may be potential targets for human breast cancer treatments.

## 2 MATERIALS AND METHODS

### 2.1 Patients and tissue samples

Fifty pairs of primary breast cancer (forty‐six cases of invasive ductal carcinoma, two cases of invasive papillary carcinoma and two cases of medullary carcinoma) and matched adjacent normal tissues were obtained between February and May 2013 at Tianjin Cancer Hospital (Table 1 and Table S1). Power analysis was used to estimate the adequacy of the sample size. No gender‐based or ethnic‐based differences were present. Clinical and clinicopathological classifications and stage were determined according to the American Joint Committee on Cancer (AJCC) criteria. The histologic grade was determined according to the Elston‐Ellis modification of the Scarff‐Bloom‐Richardson (SBR) system. All procedures involving human participants were in accordance with the ethical standards outlined in the 1964 Declaration of Helsinki and were approved by the Research Ethics Committee. Informed consent was obtained from all patients, and the study was approved by the local ethics board.

**Table 1:** The relationship between miR‐34b/c‐5p and NK1R expression and Clinicopathological parameters in breast cancer

Clinicopathological parameters	Number of cases	Expression of miR‐34b‐5p (mean ± SD)	*P*	Expression of miR‐34c‐5p (mean ± SD)	*P*	Expression of NK1R‐Tr (mean ± SD)	*P*	Expression of NK1R‐FL (mean ± SD)	*P*
Age (y)									
<50	28	1.61 ± 0.87	0.144	1.37 ± 0.80	0.228	5.72 ± 2.60	0.241	2.43 ± 2.04	0.273
≥50	22	1.95 ± 0.67		1.62 ± 0.57		4.96 ± 1.68		3.15 ± 2.55	
Mean	49.8								
TNM stage									
I, II	35	1.93 ± 0.69	0.016*	1.63 ± 0.62	0.026*	4.95 ± 1.27	0.034*	2.55 ± 1.87	0.372
III	15	1.35 ± 0.91		1.14 ± 0.82		6.41 ± 3.50		3.19 ± 3.07	
Lymph node status									
Negative	25	1.93 ± 0.73	0.123	1.55 ± 0.57	0.494	4.89 ± 1.26	0.125	2.01 ± 1.30	0.021*
Positive	25	1.58 ± 0.84		1.41 ± 0.83		5.87 ± 2.88		3.47 ± 2.80	
ER									
Negative	12	1.87 ± 0.48	0.581	1.53 ± 0.52	0.771	4.23 ± 1.32	0.041*	2.64 ± 1.53	0.864
Positive	38	1.72 ± 0.88		1.46 ± 0.77		5.75 ± 2.38		2.77 ± 2.49	
PR									
Negative	19	1.80 ± 0.70	0.791	1.62 ± 0.77	0.265	4.58 ± 2.51	0.047*	3.14 ± 2.67	0.338
Positive	31	1.73 ± 0.87		1.39 ± 0.67		5.88 ± 1.96		2.50 ± 2.02	
HER‐2									
Negative	31	1.92 ± 0.73	0.065	1.69 ± 0.65	0.006**	4.97 ± 1.40	0.101	3.04 ± 2.56	0.240
Positive	19	1.49 ± 0.85		1.13 ± 0.69		6.05 ± 3.13		2.25 ± 1.69	
Ki‐67 (%)									
Negative	12	2.25 ± 0.87	<0.001**	2.03 ± 0.81	0.002**	4.12 ± 1.85	0.025*	2.92 ± 3.24	0.767
Positive	38	1.52 ± 0.62		1.31 ± 0.59		5.78 ± 2.24		2.69 ± 1.94	

a *P* < 0.05

b *P* < 0.01.

### 2.2 Quantitative reverse transcription‐PCR analysis

Total RNA was extracted using the TRIzol (Invitrogen, Carlsbad, CA, USA). MicroRNA from frozen tissues was extracted using the mirVana miRNA Isolation Kit (Ambion, Austin, TX, USA), according to the manufacturer's instructions. Reverse transcription was performed using a First Strand cDNA Synthesis kit (Invitrogen). The primers for miR‐34a‐3p, miR‐34a‐5p, miR‐34b‐3p, miR‐34b‐5p, miR‐34c‐3p, miR‐34c‐5p and U6 detection assays were purchased from RiboBio (Guangzhou, China), and the primers sequences for NK1R‐FL, NK1R‐Tr and β‐actin have been previously published.^22^ All reactions were performed in a 20 μL reaction volume in triplicate. Data were assessed using the 2^−△△Ct^ method. PCR amplification was performed as follows: 15 s at 95°C, 15 s at 51‐58°C (depending on the type of gene) and 45 s at 72°C for 40 cycles.

### 2.3 Cell culture

Human breast cancer cell lines (MDA‐MB‐231, MCF‐7, T47D and SK‐BR‐3) and a human embryonic kidney cell lines (HEK‐293 T) were purchased from the American Type Culture Collection (ATCC, Manassas, VA, USA). The nontumorigenic mammary epithelial cell line HBL‐100 was purchased from the Shanghai Institute of Cell Biology, Chinese Academy of Sciences. Cells were cultured in DMEM/F12 medium (Hyclone, Logan, UT, USA) supplemented with 10% foetal bovine serum (FBS; Hyclone) at 37℃ in a humidified atmosphere containing 5% CO_2_. SP (MCE, Shanghai, China) was added to all cultures at a final concentration of 100 nmol/L. A stock solution of 10 mmol/L aprepitant (Santa Cruz, CA, USA) was prepared by dissolving the compound in 0.1% sterile dimethyl sulfoxide (DMSO), divided into aliquots and then stored at −20°C until used. Different concentrations of aprepitant (5‐70 μmol/L) were evaluated to determine the IC_50_. Cells were treated with relevant amounts of the aprepitant stock solution to attain concentrations of 5 and 10 μmol/L and cells were treated with the corresponding concentration of DMSO as a negative control.

### 2.4 Cell transfection and luciferase reporter assay

The specific siRNA sequences of NK1R and negative controls were purchased from Invitrogen and miR‐34a‐3p, miR‐34a‐5p, miR‐34b‐3p, miR‐34b‐5p, miR‐34c‐3p, miR‐34c‐5p mimics, inhibitors, mimic control and inhibitor control were purchased from RiboBio. Cells were cultured in six‐well plates until they were in the logarithmic growth phase and then transfected with either 100 pmol/well siNK1R and miRNA mimics or 400 pmol/well miRNA inhibitors using Lipofectamine 2000 (Invitrogen) according to the manufacturer's protocol. After a 48 hour transfection, the cells were harvested for further analysis.

MDA‐MB‐231, SK‐BR‐3, T47D and HEK‐293 T cells in the logarithmic phase were co‐transfected with the reporter plasmid NK1R‐FL‐3’UTR‐WT, miR‐34b/c‐5p mimic or mimic control according to the manufacturer's instructions. Site‐specific mutants of the 3’UTR reporters for NK1R‐FL were also tested. To investigate whether SP induced NK1R signalling, SP treatment was coupled to luciferase activity, which was measured. Briefly, cells were transfected with the reporter plasmid PNK1R‐luc. Twenty‐four hour later, the transfected cells were seeded in a 96‐well plate at a density of 30 000 and cultured for another 24 hour. The cells were exposed to 100 nmol/L SP for 8 h at 37°C. Untreated cells were used as a control. Luciferase activities were measured using a luciferase assay system.

### 2.5 Western blotting

After SP treatment for 48 h, cells were rapidly washed with chilled 1 × PBS. Cultured cells were removed using a cell scraper, and proteins were isolated using lysis buffer (50 mmol/L Tris‐HCl, pH 7.4, 1% NP‐40, 0.25% Na‐deoxycholate, 150 mmol/L NaCl, 1.0 mmol/L EDTA, 1.0 mmol/L PMSF and 1 × protease inhibitor cocktail from Roche, Guangzhou, China). Protein extracts were resolved by 10% SDS‐PAGE and transferred to polyvinylidene fluoride membranes (Millipore, Boston, MA, USA). After being blocked with 5% BSA, the blots were probed with the following primary antibodies: p‐ERK (1:1000), ERK1/2 (1:1000), p‐AKT (Ser 473; 1:1000), AKT (1:1000), cyclin B1 (1:1000), CDC23 (1:1000) from Cell Signaling Technology (Boston, MA, USA), NK1R (1:250) from R&D Systems (Minneapolis, MN, USA) and β‐actin (1:5000) from Sigma‐Aldrich. After three washes, the membranes were incubated at 4°C and then incubated with the appropriate HRP‐linked secondary antibodies (Santa Cruz) and then blots were developed using ECL (Millipore). β‐Actin was used as internal control.

### 2.6 CCK‐8 assay

Cell suspensions were seeded in 96‐well plates at an initial density of 10^3^ per well and incubated for 24 hour. After incubation in serum‐free medium for 24 hour, cells were treated with or without SP To determine the effect of an NK1R antagonist on breast cancer cell proliferation, cells were treated with aprepitant at the indicated dose. Afterwards, at each time point, cells were stained with 100 μL of CCK‐8 solution (Dojindo, Kumamoto, Japan) for 2 hours in a humidified atmosphere (37°C, 5% CO_2_) in the dark, followed by replacement with fresh culture medium. The absorbance of each sample was measured at 450 nm using a universal microplate reader (Bio‐Tek). All experiments were performed in triplicate.

### 2.7 Colony formation

Cells were plated in 35‐mm plates (5 × 10^2^ cells per plate) after treatment with SP or aprepitant and were cultured in a humidified atmosphere (37°C, 5% CO_2_) for 15 days. The colonies were fixed with methanol and stained with 1% crystal violet for 30 s after fixation with 10% formaldehyde for 5 minutes. Colonies with >50 cells were scored.

### 2.8 Cell cycle analysis

Cells were treated with or without SP or chemical inhibitors as indicated and then processed for cell cycle analysis using flow cytometry. Briefly, cells were fixed in 75% ethanol at 4°C overnight. After being centrifuged at 200 ***g***, RNase A (20 μg/mL final concentration) and propidium iodide staining solution (50 μg/mL final concentration) were added to the cells, and the cells were then incubated for 30 minutes at 37°C in the dark. Fifty thousand cells were analysed using a FACScan flow cytometer (Becton Dickinson, Franklin lake, NJ, USA). ModFit LT 3.1 trial cell cycle analysis software was used to determine the percentage of cells in the different phases of the cell cycle.^30^

### 2.9 Apoptosis assay

Commercial Caspase Activity Assay kits were used to assess the activities of caspase‐3 (Promega, Madison, WI, USA) and caspase‐8 and caspase‐9 (Beyotime, Shanghai, China). An equal amount of total protein extract was incubated at 37°C overnight with either Ac‐DETD‐PNA for the caspase‐3 assay, Ac‐IETD‐pNA for the caspase‐8 assay or Ac‐LEHD‐pNA for the caspase‐9 assay. Release of pNA was determined by absorbance at 405 nm. The relative activity of caspases was calculated as follows: caspase activity = (mean experimental absorbance/mean control absorbance) × 100%. All procedures were performed three times.

Apoptosis was detected by fluorescein isothiocyanate (FITC)‐Annexin V/propidium iodide (PI) double staining (Invitrogen). After 48 h of treatment with SP or aprepitant, the cells were washed twice with PBS. The cells were labelled with FITC‐Annexin V and PI for 10 minutes in the dark and cellular fluorescence was measured using a FACScan flow cytometer (Becton Dickinson). Each experiment was performed in triplicate.^31^

### 2.10 Nude mice assay

Female BALB/c‐nu mice (4‐5 weeks of age, 18‐20 g) were purchased from the Speyfor Biotechnology Company (Beijing, China) and were housed in barrier facilities on a 12 hour light/dark cycle. All experimental procedures were approved by the Animal Ethical Committee of Tianjin Medical University Cancer Institute and Hospital. The use of mice for this study was reviewed and approved by the Institutional Animal Care and Use Committee, in accordance with the China National Institutes of Health guidelines. The BALB/c nude mice were randomly divided into ten groups. Cells overexpressing miR‐34b/c‐5p, NK1R silencing and their controls were resuspended in PBS at a concentration of 2.5 × 10^6^ cells in 0.25 mL and injected into the mammary fat pads of female athymic mice. One week after implantation when the tumour became palpable at the size of ~2 mm in diameter, intratumour injection with 50 μg/kg of SP dissolved in saline daily. The other group was treated with saline. Tumours were examined and measured twice weekly. The length, width and thickness measurements were obtained using calipers and the tumour volumes were calculated. On day 24, the animals were euthanized and the tumours were excised and weighed.

### 2.11 Immunohistochemical staining

Immunohistochemistry was performed on paraformaldehyde‐fixed paraffin sections. The primary antibody of Ki67 (1:100) was purchased from Beijing Zhongshan Biotechnology Inc (Beijing, China). Immunohistochemistry was performed as previously reported.^32^ The percentage of positive cells was graded as per the following criteria: 0, less than 10%; 1, 10%‐30%; 2, 30%‐50%; 3, more than 50%.^33^

### 2.12 Statistical analysis

All results were performed using SPSS software for Windows version 23.0 (IBM, SPSS, Chicago, IL, USA). Statistically significant differences between groups were determined using the Student's two‐tailed *t* test or the two‐way ANOVA test. The association between pairs of variables was determined using Spearman order correlations. The IC_50_ of aprepitant was calculated using the regression straight line function based on the least squares technique. All experiments for cell culture were performed at least in triplicate. The results were expressed as the mean ± SD. In all cases, *P *< 0.05 was considered statistically significant.

## 3 RESULTS

### 3.1 MiR‐34b/c‐5p regulates the expression of NK1R‐FL and NK1R‐Tr in breast cancer cells

**Figure 1:**
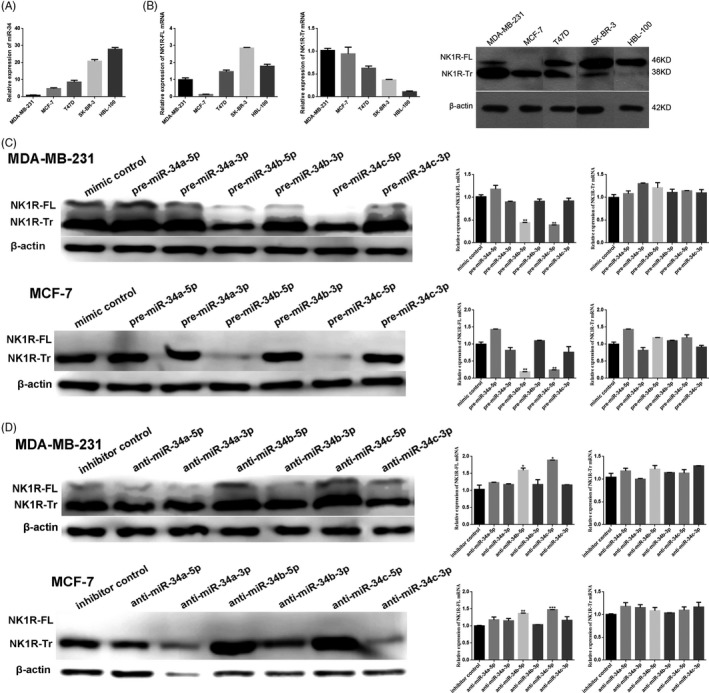
MiR‐34b/c‐5p regulates NK1R‐FL and NK1R‐Tr in breast cancer cells. A, MiR‐34 expression in breast cancer cells. Relative expression was normalized to U6 expression. B, NK1R‐FL and NK1R‐Tr mRNA and protein expression in breast cancer cells. β‐Actin was used as a loading control. C, NK1R‐FL and NK1R‐Tr expression after treatment with the mimic control or pre‐miR‐34a/b/c‐3p or pre‐miR‐34a/b/c‐5p in MDA‐MB‐231 (top) and MCF‐7 (bottom) cells. Relative expression was normalized to β‐actin expression. NK1R‐FL and NK1R‐Tr protein expression and NK1R‐FL mRNA expression in pre‐miR‐34b/c‐5p‐treated cells were significantly downregulated compared with untreated cells, pre‐miR‐34a/b/c‐ 3p‐treated cells and pre‐miR‐34a‐5p‐treated cells. The results show the mean ± SD of triplicates. D, NK1R‐FL and NK1R‐Tr expression after treatment with an inhibitor control or anti‐miR‐34a/b/c‐3p or anti‐miR‐34a/b/c‐5p in MDA‐MB‐231 (top) and MCF‐7 (bottom) cells. Relative expression was normalized to β‐actin expression. NK1R‐FL and NK1R‐Tr protein expression and NK1R‐FL mRNA expression in anti‐miR‐34b/c‐5p‐treated cells were significantly higher than that in untreated cells, anti‐miR‐34a/b/c‐3p cells and anti‐miR‐34a‐5p cells. The results show the mean ± SD of three independent experiments. *P < 0.05; **P < 0.01; ***P < 0.001

The expression levels of miR‐34, NK1R‐FL and NK1R‐Tr were detected in different breast cancer cell lines, including MDA‐MB‐231, MCF‐7, T47D, SK‐BR‐3 and the nontumorigenic mammary epithelial cell line HBL‐100 (Figure 1A,B). Consistent with our previous reports,^22^, ^23^ the MDA‐MB‐231, MCF‐7 and T47D cell lines had relatively high NK1R‐Tr expression and low NK1R‐FL and miR‐34 expression,^34^, ^35^ while HBL‐100 expressed only NK1R‐FL. To examine the correlation of miR‐34 and NK1R in breast cancer, MDA‐MB‐231 and MCF‐7 cells, which express high level of NK1R‐Tr, were transfected with miR‐34a‐3p, miR‐34a‐5p, miR‐34b‐3p, miR‐34b‐5p, miR‐34c‐3p, miR‐34c‐5p mimics and inhibitors. Overexpression of miR‐34b‐5p and miR‐34c‐5p in MDA‐MB‐231 and MCF‐7 cells resulted in a significant reduction in NK1R‐Tr protein expression but not mRNA expression, while overexpression of miR‐34b‐5p and miR‐34c‐5p significantly downregulated both the mRNA and protein expression of NK1R‐FL (*P *< 0.05). Conversely, cells transfected with miR‐34b‐5p and miR‐34c‐5p inhibitors exhibited high levels of NK1R‐FL and NK1R‐Tr (*P *< 0.05). However, changes in the expression of miR‐34a‐3p, miR‐34a‐5p, miR‐34b‐3p and miR‐34c‐3p had no effect on NK1R‐FL and NK1R‐Tr expression (*P *> 0.05, Figure 1C,D).^36^ To further explore the regulation of miR‐34b‐5p and miR‐34c‐5p by NK1R, dual‐luciferase reporter systems were established in MDA‐MB‐231, SK‐BR‐3, T47D and HEK‐293 T cells (Figure S1). Transient co‐transfection with the NK1R‐FL reporter plasmid and pre‐miR‐34b‐5p or pre‐miR‐34c‐5p significantly reduced luciferase activity compared with the negative controls in HEK‐293 T, MDA‐MB‐231, SK‐BR‐3 and T47D cells (*P *< 0.05). However, the activity of the reporter vector containing a mutant 3′UTR sequence was unaffected by simultaneous transfection with pre‐miR‐34b‐5p or pre‐miR‐34c‐5p (*P *> 0.05). These results indicated that NK1R‐FL might be the target of miR‐34b/c‐5p, and that miR‐34b/c‐5p may reduce NK1R‐Tr expression through some intermediaries.

### 3.2 MiR‐34b/c‐5p and NK1R‐Tr expression is associated with the clinicopathological parameters of breast cancer

**Figure 2:**
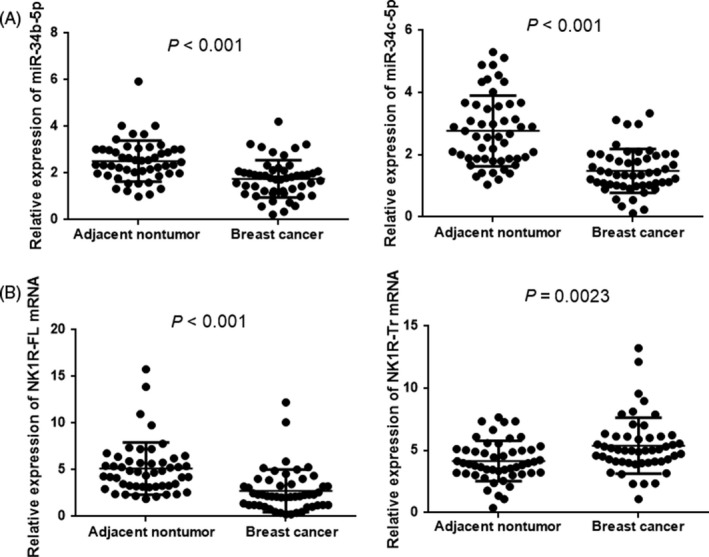
MiR‐34b/c‐5p expression is inversely correlated with NK1R‐Tr expression in breast cancer tissues. A, Expression of miR‐34b‐5p (left) and miR‐34c‐5p (right) in 50 pairs of breast cancer and adjacent nontumor tissues. Relative expression was normalized to the U6 level from the same sample. B, Expression of NK1R‐FL (left) and NK1R‐Tr (right) mRNA in the same samples. Relative expression was normalized to β‐actin expression. Values are expressed as the mean ± SD of three independent experiments

To investigate the relationship between the expression of miR‐34b/c‐5p and NK1R and the clinicopathological parameters of breast cancer, the expression levels of miR‐34b/c‐5p, NK1R‐FL and NK1R‐Tr were examined by qRT‐PCR in 50 paired samples of breast cancer and matched adjacent nontumour tissues. Significant differences in the expression of miR‐34b/c‐5p and NK1R were observed between tumour and adjacent nontumour tissues (Figure 2A,B). Among them, miR‐34b/c‐5p and NK1R‐FL were significantly overexpressed in normal breast tissues (*P *< 0.001).^35^ Conversely, NK1R‐Tr expression was significantly overexpressed in breast cancer tissues (*P *= 0.0023).^22^ As shown in Table 1, we quantified miR‐34b/c‐5p and NK1R expression in tumour samples from 50 breast cancer patients. MiR‐34b/c‐5p expression was much lower in the advanced clinical stages than in the early clinical stages (*P *= 0.016, *P *= 0.026), whereas NK1R‐Tr expression was significantly higher in the advanced clinical stages (*P *= 0.034) NK1R‐FL showed no significant differences among the groups (*P *= 0.372). NK1R‐Tr was significantly overexpressed in oestrogen receptor (ER)*−* and progesterone receptor (PR)*−* positive tumours (*P *= 0.041 and *P *= 0.047, respectively); however, there was no difference in miR‐34b/c‐5p expression (miR‐34b‐5p, *P *= 0.581, *P *= 0.791; miR‐34c‐5p, *P *= 0.771, *P *= 0.265) or in NK1R‐FL expression (*P *= 0.864, *P *= 0.338). Strikingly, statistical analyses indicated that miR‐34b/c‐5p and NK1R‐Tr expression, but not NK1R‐FL expression, were strongly associated with Ki67 levels. There was no significant correlation between miR‐34b/c‐5p and NK1R‐Tr expression and age and lymph node status (Table 1, Table S1). Interestingly, Pearson correlation analyses revealed a significant positive correlation between the miR‐34b‐5p and miR‐34c‐5p expression levels (*r* = 0.762, *P *< 0.001, Figure S2A). MiR‐34b‐5p expression was inversely correlated with NK1R‐Tr expression (*r* = −0.538, *P *< 0.001), and miR‐34c‐5p expression was inversely correlated with NK1R‐Tr expression (*r* = −0.529, *P *< 0.001, Figure S2B); however, there was no significantly correlation between miR‐34b/c‐5p and NK1R‐FL (miR‐34b‐5p, *r* = 0.120, *P *= 0.406; miR‐34c‐5p, *r* = 0.087, *P *= 0.549). Together, these observations support the hypothesis that NK1R‐Tr is closely associated with miR‐34b/c‐5p.

### 3.3 MiR‐34b/c‐5p and NK1R regulate the proliferation of breast cancer cells

**Figure 3:**
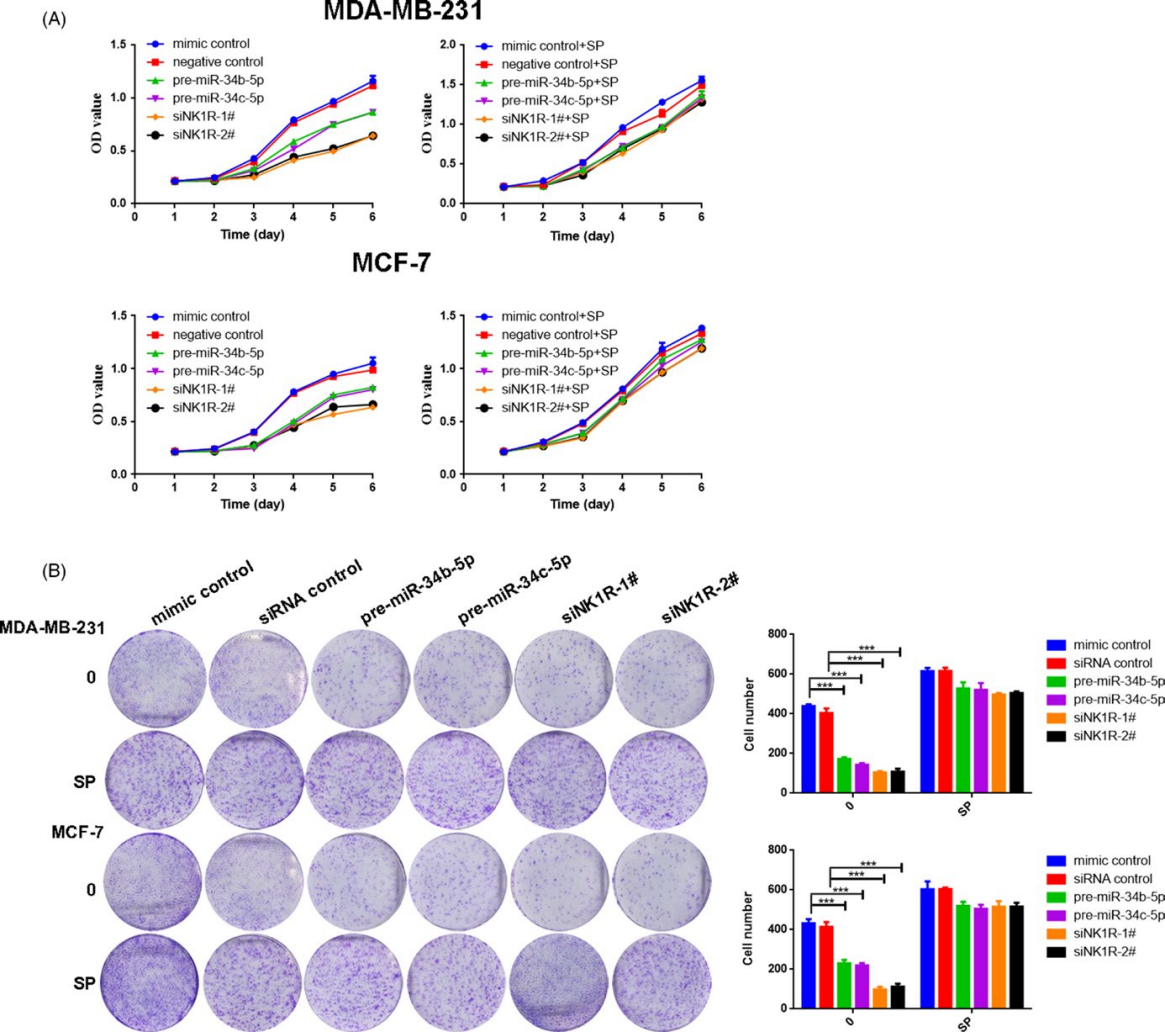
MiR‐34b/c‐5p and NK1R are essential for breast cancer cell proliferation. A, CCK‐8 test. B, Colony formation assay were used to investigate the regulation of miR‐34b/c‐5p and NK1R with or without SP treatment. Values are expressed as the mean ± SD of three independent experiments. ***P < 0.001

We found that SP was able to induce NK1R signalling in MDA‐MB‐231 and MCF‐7 cells in comparison with control groups as indicated by our luciferase assay (*P *< 0.001, Figure S3). To analyse the effect of miR‐34b/c‐5p and NK1R on cell proliferation, MDA‐MB‐231 and MCF‐7 cells were transfected with miR‐34b/c‐5p mimic or NK1R‐siRNA and their corresponding controls before treatment with or without SP MDA‐MB‐231 and MCF‐7 cells overexpressing miR‐34b/c‐p had significantly suppressed proliferation rates compared with the control cells, as analysed by the CCK‐8 assay (*P *< 0.05). SP treatment significantly impaired this effect (Figure 3A). These results were further confirmed by a colony formation assay (Figure 3B). In addition, we also investigated whether aprepitant had potential antitumour efficacy (Figure S4A,B), and we determined the IC_50_ growth inhibition concentration of aprepitant needed for tumour cell lines (Table S2). Based on our previous study, NK1R‐FL and NK1R‐Tr have opposing roles in cell proliferation, and our present results further support the notion that miR‐34b/c‐5p inhibits cell proliferation by regulating NK1R‐Tr

### 3.4 MiR‐34b/c‐5p and NK1R regulate the G2/M phase transition in breast cancer

**Figure 4:**
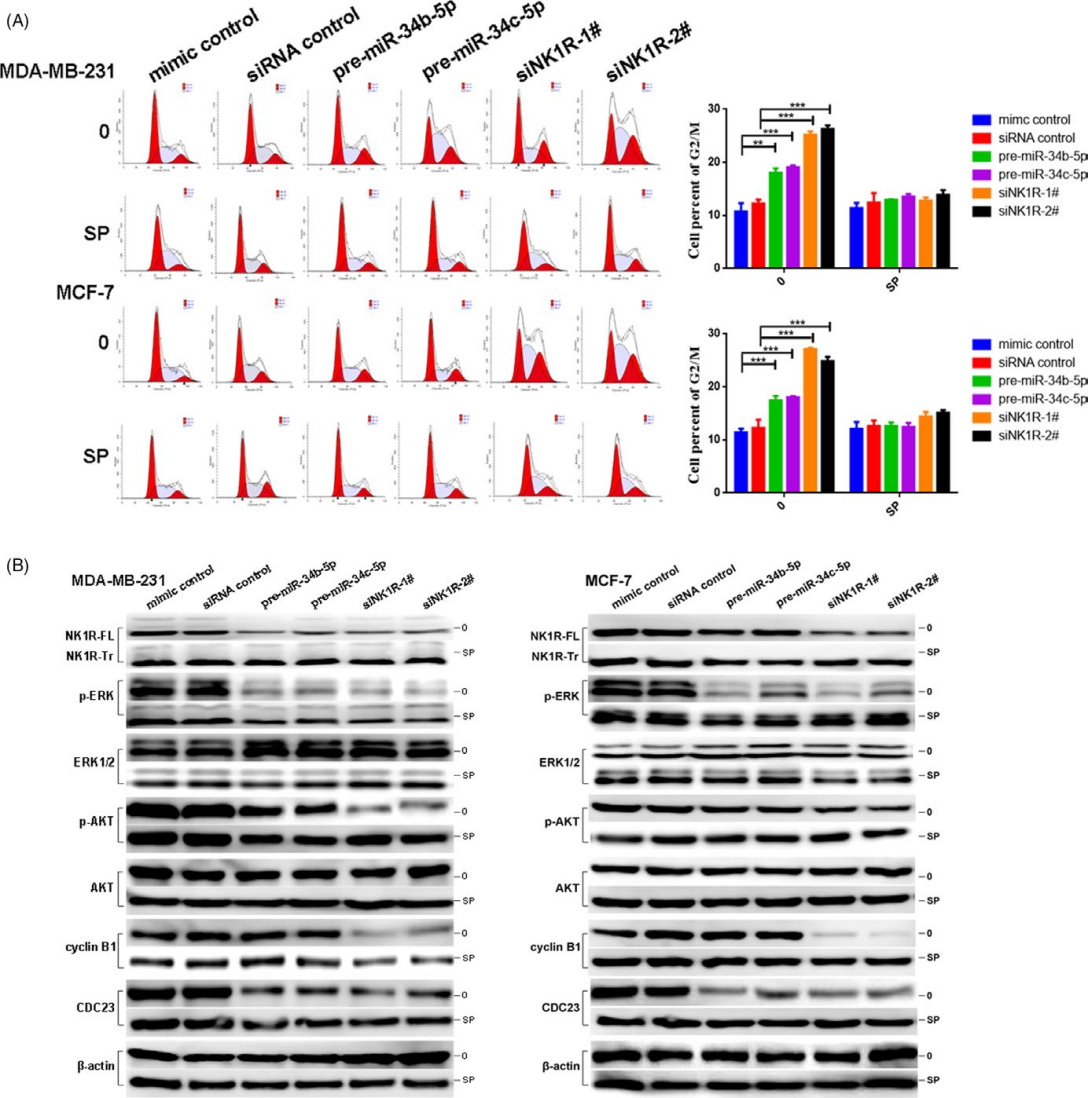
MiR‐34b/c‐5p and NK1R regulate the G2/M phase transition in breast cancer cells. A, Flow cytometry analysis of negative control and miR‐34b/c‐5p‐overexpressing and NK1R‐silenced cells with or without SP treatment (left). Quantification of cells in the G2/M phase cells (right). B, Western blot analysis of NK1R, phosphorylated ERK (p‐ERK), ERK1/2, phosphorylated AKT (Ser 473, p‐AKT), AKT, cyclin B1 and CDC23 proteins with or without SP treatment after transfection with pre‐miR‐34b/c‐5p or NK1R‐siRNA. β‐Actin was used as a loading control. Values are expressed as the mean ± SD of three independent experiments. **P < 0.01; ***P < 0.001

We conducted flow cytometry analysis to further investigate the potential mechanism of miR‐34b/c‐5p‐ and NK1R‐mediated proliferation. As shown in Figure 4A, the overexpression of miR‐34b/c‐5p or NK1R silencing in MDA‐MB‐231 and MCF‐7 cells significantly increased the percentage of cells in the G2/M phase (*P *< 0.01).^37^ Furthermore, compared with the control group, after SP treatment, the percentage of cells in the G2/M phase decreased. In addition, high doses of aprepitant had more intense effects on cell cycle arrest (*P *< 0.001, Figure S5A). NK1R regulates cell proliferation by mediating the activity of the ERK1/2 and PI3K/AKT signalling pathways. We examined the effect of miR‐34b/c‐5p and NK1R on the regulation of the phosphorylation activities of ERK and AKT. As shown in Figure 4B, the levels of p‐ERK and p‐AKT were decreased in miR‐34b/c‐5p‐overexpressing or NK1R‐silenced cells, but SP lessened their effects. Aprepitant also decreased the phosphorylation activity of ERK and AKT (Figure S5B). We also investigated the function of miR‐34b/c‐5p and NK1R on cell cycle‐related proteins. Cyclin B1 and CDC23 mRNA and protein expression were significantly reduced in NK1R‐silenced and aprepitant‐treated cells compared with control cells (*P *< 0.05; Figures S5B,C and S6). However, only CDC23, not cyclin B1, was decreased in miR‐34b/c‐5p overexpressing cells (Figure 4B). In addition, cyclin B1 and CDC23 levels increased after SP treatment. Collectively, our results indicate that miR‐34b/c‐5p induced cell cycle arrest at the G2/M phase mainly by regulating CDC23 and that NK1R mainly regulates cyclin B1 and CDC23.

### 3.5 MiR‐34b/c‐5p and NK1R regulate breast cancer cell apoptosis

**Figure 5:**
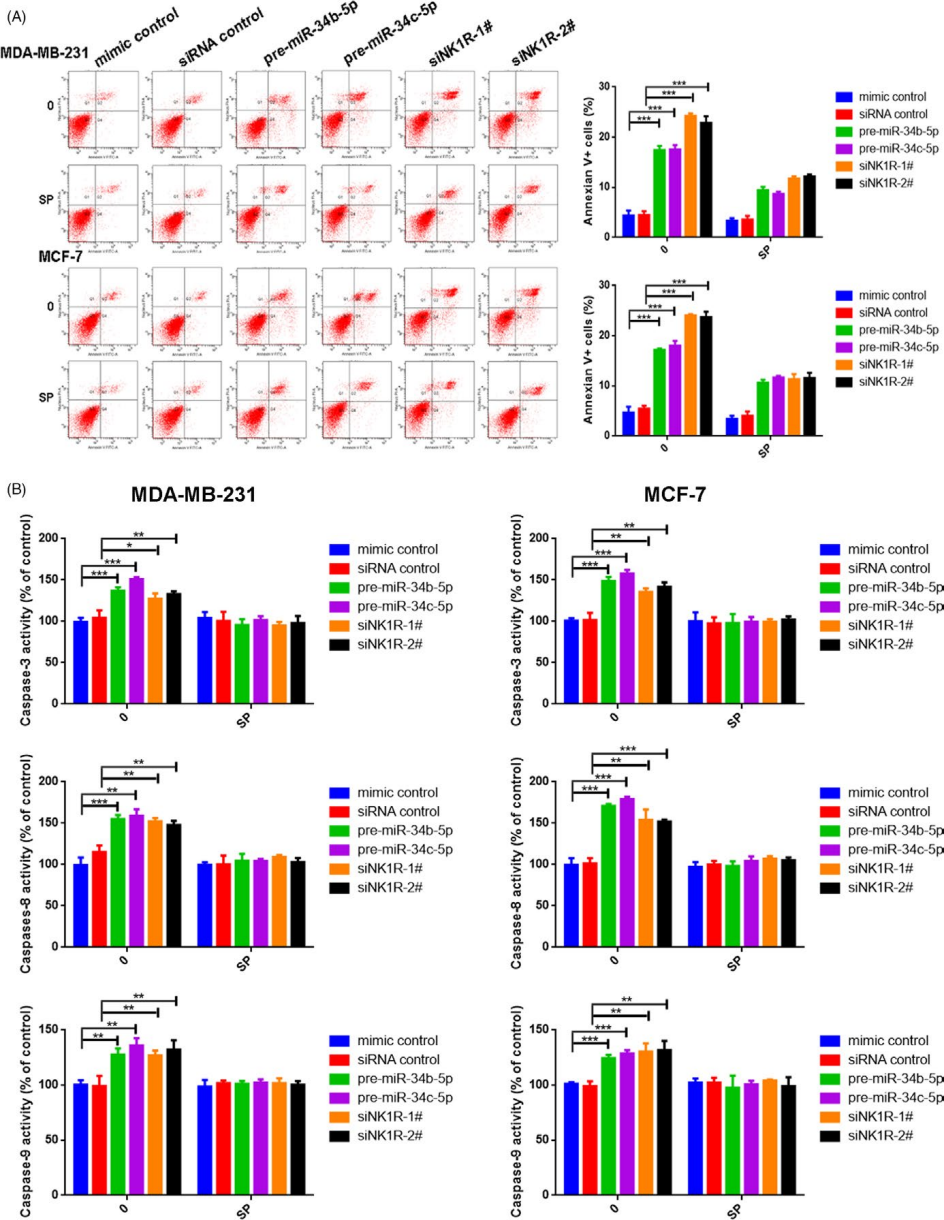
MiR‐34b/c‐5p and NK1R regulate apoptosis in breast cancer MDA‐MB‐231 and MCF‐7 cells. A, Apoptosis was evaluated after culturing MDA‐MB‐231 and MCF‐7 cells transfected with miR‐34b/c‐5p or NK1R‐siRNA, before treatment with or without SP and staining with Annexin V at 48 h. The flow cytometry profile depicts Annexin V/FITC staining on the x‐axis and PI staining on the y‐axis (left), the number represents the percentage of total apoptotic cells in each condition (right). B, Caspase‐3, caspase‐8 and caspase‐9 activities were detected in MDA‐MB‐231 (left) and MCF‐7 (right) cells transfected with miR‐34b/c‐5p or NK1R‐siRNA with or without SP treatment. The data represent the mean ± SD of three independent experiments. *P < 0.05; **P < 0.01; ***P < 0.001

We investigated the apoptosis induced by miR‐34b/c‐5p or NK1R silencing in MDA‐MB‐231 and MCF‐7 cells. Annexin V/PI staining showed that transfection with pre‐miR‐34b/c‐5p or NK1R‐siRNA induced cell apoptosis that could be recovered by SP treatment (Figure 5A). Additionally, the apoptosis rate increased as the aprepitant concentration increased (*P *< 0.001, Figure S5D). This finding was further supported by our analysis of caspase‐3, caspase‐8 and caspase‐9 activity. Transfection with pre‐miR‐34b/c‐5p or NK1R‐siRNA or aprepitant treatment significantly increased caspase‐3, caspase‐8 and caspase‐9 activity in either MDA‐MB‐231 or MCF‐7 cells (Figure 5B, Figure S5E), suggesting the activation of both extrinsic and intrinsic pathways for apoptosis; however, SP treatment reduced the activity of caspase‐3, caspase‐8 and caspase‐9 in cells transfected with pre‐miR‐34b/c‐5p or siRNA‐NK1R (Figure 5B). The induction of apoptosis by miR‐34b/c‐5p or NK1R‐silencing occurred through an intrinsic apoptosis pathway.

### 3.6 *Transfection of miR‐34b/c‐5p or downregulation of NK1R inhibits the tumorigenicity of breast cancer cells* in vivo

**Figure 6:**
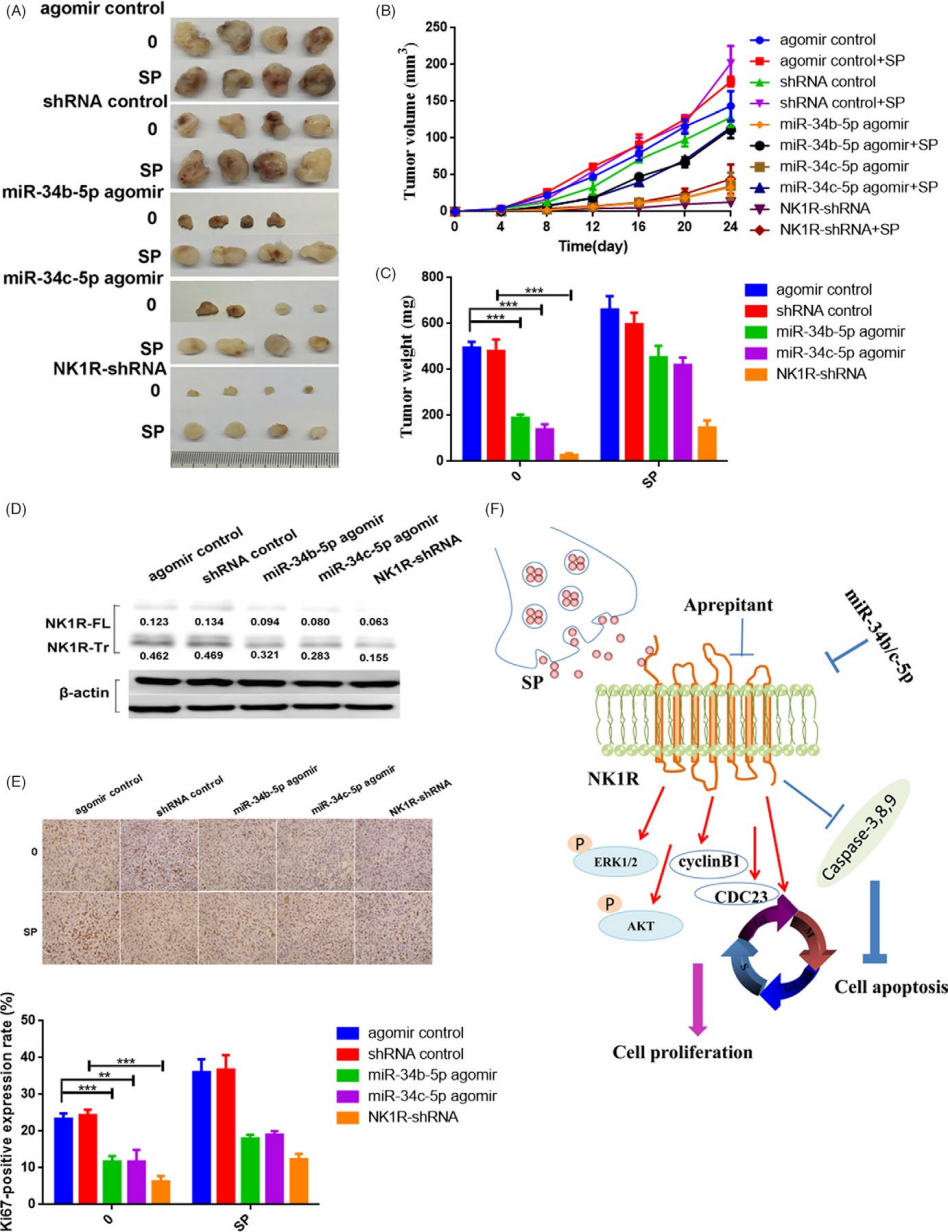
Overexpression of miR‐34b/c‐5p and knockdown of NK1R inhibit the tumorigenicity of breast cancer cells in vivo. A, Representative images of tumour growth. B, Tumour volume growth curves. C, Mean tumour masses 24 days after inoculation. All data are presented as the mean ± SD. D, Proteins extracted from tumour tissues were analysed by IB using anti‐NK1R antibody, the densitometric evaluation of NK1R‐Tr was under the band. E, The immunohistochemical stain for Ki67 is shown (×400, top). Quantification of Ki67 expression (bottom). F, Proposed regulation model of miR‐34b/c‐5p and SP‐NK1R on cell proliferation and apoptosis in breast cancer. **P < 0.01; ***P < 0.001

Because the overexpression of miR‐34b/c‐5p or NK1R‐silencing inhibited breast cancer cell growth in vitro, we proceeded to evaluate their effects on tumour formation in vivo. MDA‐MB‐231 cells overexpressing miR‐34b/c‐5p, NK1R silencing or their controls were inoculated into nude mice. As shown in Figure 6A and B, tumours transfected with the miR‐34b/c‐5p agomir grew significantly slower than control tumours (*P *< 0.01), and NK1R‐silencing xenografted tumours grew much slower than corresponding controls (*P *< 0.01), which was consistent with our in vitro cell proliferation results. Almost four weeks later, the weights of the tumours in mice transplanted with cells transfected with miR‐34b/c‐5p or NK1R‐shRNA were significantly reduced compared with mice transplanted with control cells (*P *< 0.01, Figure 6C). In addition, SP treatment recovered the inhibition of overexpression of miR‐34b/c‐5p or NK1R knockdown on tumour size and tumour weight. We also examined Ki67 and NK1R expression in xenografted tumours. As shown in Figure 6D and E, NK1R‐Tr expression and immunohistochemistry staining of the proliferation marker Ki67 were significantly down‐regulated following miR‐34b/c‐5p overexpression or NK1R knockdown. Consistent with the in vitro results, SP was able to rescue the expression inhibition of NK1R‐Tr and Ki67 by miR‐34b/c‐5p overexpression or NK1R knockdown in vivo. These results suggest that miR‐34b/c‐5p and NK1R play an important role in reducing the tumorigenicity of breast cancer cells in vivo.

## 4 DISCUSSION

These data show that miR‐34 and NK1R‐FL expression was downregulated and that NK1R‐Tr expression was upregulated in breast cancer cell lines. MiR‐34b/c‐5p overexpression was significantly correlated with NK1R‐FL and NK1R‐Tr expression. Low miR‐34b/c‐5p expression and high NK1R‐Tr expression were also significantly correlated with the clinicopathological characteristics of breast cancer, such as clinical stage and Ki67 expression, but not NK1R‐FL, indicating that the upregulation of miR‐34b/c‐5p and the downregulation of NK1R‐Tr may suppress the development of breast cancer. We also observed high expression of NK1R‐Tr in ER‐ or PR‐positive patients, which was consistent with previous data.^35^, ^38^, ^39^ Moreover, a Pearson correlation analysis showed a significant negative correlation between miR‐34b/c‐5p and NK1R‐Tr expression in breast cancer patients, which provides further evidence for the regulation of NK1R‐Tr by miR‐34b/c‐5p. In humans, miR‐34 s are encoded by two different genes: miR‐34a is encoded by its own transcript and miR‐34b and miR‐34c share a common primary transcript.^40^, ^41^ In this study, the close association of miR‐34b‐5p and miR‐34c‐5p is consistent with the current hypothesis that miRNAs in the same chromosomal position play a similar role in tumorigenesis. Interestingly, miR‐34b‐5p and miR‐34c‐5p reduced NK1R‐FL expression by binding to the 3’UTR of NK1R‐FL in MDA‐MB‐231, SK‐BR‐3, T47D and HEK‐293 T cells. These findings provide strong evidence that miR‐34b‐5p, miR‐34c‐5p and NK1R play important roles in the progression of breast cancer; thus, the roles of NK1R‐Tr and NK1R‐FL in breast cancer proliferation were also investigated.

MiR‐34 s serve as breast cancer suppressors by regulating hundreds of downstream targets.^35^, ^42^, ^43^ From our previous studies, the two isoforms of NK1R, NK1R‐FL and NK1R‐Tr, are involved in different biological actions in breast cancer: here, we show that NK1R‐Tr was markedly upregulated and that NK1R‐FL was markedly downregulated in breast cancer cells and tissues. Therefore, further experiments are necessary to investigate the regulation of miR‐34b/c‐5p to NK1R. Indeed, our CCK‐8 and clone formation tests showed that the effect of miR‐34b/c‐5p on cell proliferation appears to be similar to that of NK1R silencing and endogenous peptide agonists of NK1R, SP, could rescue this effect partly. Moreover, aprepitant treatment also had this inhibitory effect. Consistent with other reports, the upregulation of miR‐34b/c‐5p induced G2‐M‐phase arrest.^37^ The downregulation of NK1R and aprepitant treatment also induced G2‐M‐phase arrest, while, SP treatment did not. Furthermore, the proliferation mechanism of miR‐34b/c‐5p and NK1R was tightly linked to the regulation of cell cycle regulators. CDC23, among others, is a direct target of miR‐34b/c,^37^, ^44^ and our results confirmed that miR‐34b/c‐5p reduced its expression level in breast cancer. NK1R silencing downregulated the mRNA and protein expression of both cyclin B1 and CDC23 and SP treatment impaired the effect, which indicates that miR‐34b/c‐5p and NK1R arrest the cell cycle through two different signalling pathways.

Consistent with the inhibitory effects of miR‐34b/c‐5p overexpression and NK1R silencing on breast cancer cell growth, their ability to induce apoptosis was also shown in our Annexin V analysis. As expected, aprepitant treatment had a similar effect on inducing cell apoptosis, well SP had the opposite effect. Furthermore, the overexpression of miR‐34b/c‐5p or NK1R silencing in MDA‐MB‐231 and MCF‐7 cells increased the activity of all 3 caspases (caspase‐3 effectors, caspase‐8 and caspase‐9 initiators), while SP treatment reversed their effect, which suggests that the induction of apoptosis by miR‐34b/c‐5p or NK1R not only occurs via the intrinsic pathway but also occurs extrinsically. In addition, aprepitant treatment also increased caspase‐3, caspase‐8 and caspase‐9 activities, suggesting that extrinsic apoptosis may occur while intrinsic might be secondary.

In line with the in vitro data, our in vivo data also demonstrated that overexpression of miR‐34b/c‐5p resulted in a significant decrease in expression of NK1R‐Tr and Ki67, as well as an inhibition of tumour growth, and SP was able to rescue the inhibition effect. Knockdown of NK1R resulted in a significant decrease in Ki67 expression, as well as an inhibition of tumour growth. Interestingly, SP could also rescue this inhibition partly. Based on our previous results and other studies,^23^, ^45^, ^46^, ^47^, ^48^ SP may rescue this effect through regulating some intermediaries positively, such as TGF‐β1, EGFR and HER‐2. Together, our data suggest that miR‐34b/c‐5p and NK1R may be potential targets for breast cancer therapy.

Importantly, this study showed that the impact of NK1R suppression on the growth and apoptosis of MDA‐MB‐231 cells was the same as that in MCF‐7 cells, which overexpress NK1R‐Tr These data suggest that the regulation of NK1R‐Tr by miR‐34b/c‐5p may be the main mechanism for inhibiting breast cancer cell growth.

In summary, our data suggest that miR‐34b/c‐5p is a powerful tumour suppressor. Furthermore, the inhibition of breast cancer cell growth by miR‐34b/c‐5p is mainly achieved by targeting NK1R‐Tr (Figure 6F), but the specific regulatory mechanism needs further study.

## CONFLICT OF INTEREST

The authors declare no competing financial interests or conflicts in relation to the work described.

## AUTHOR CONTRIBUTIONS

Study design: YLZ, LFZ and LSW; Data Collection: DD, ZYW and WJ; Data analysis: MY, FZ; Manuscript preparation: LFZ, RFN and YLZ; all authors reviewed and approved manuscript.
