# Supplementary figures and images for: MiR‐34b/c‐5p and the neurokinin‐1 receptor regulate breast cancer cell proliferation and apoptosis
Source: Cell Prolif. 2018 Oct 17;52(1):e12527. doi: 10.1111/cpr.12527 (PMC6430481; doi:10.1111/cpr.12527)

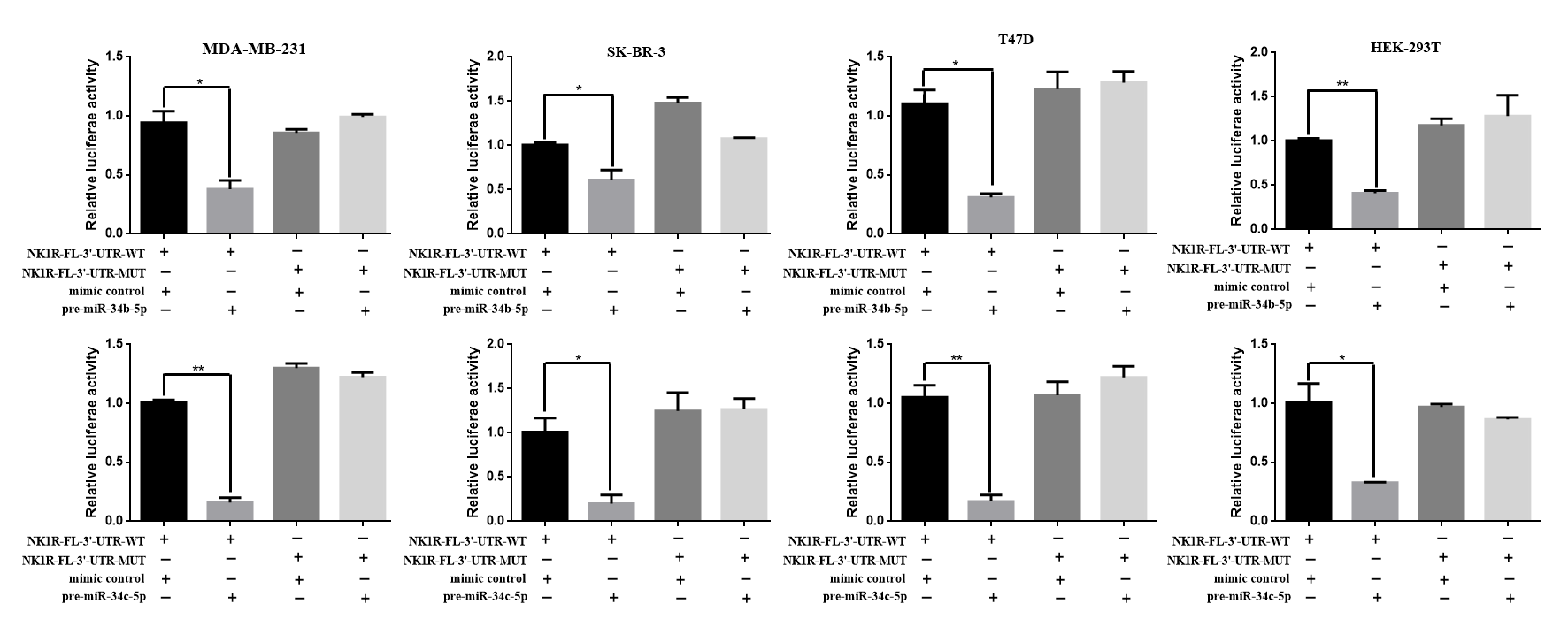

Supplement: Supplementary file 1 [file CPR-52-e12527-s001.tif]

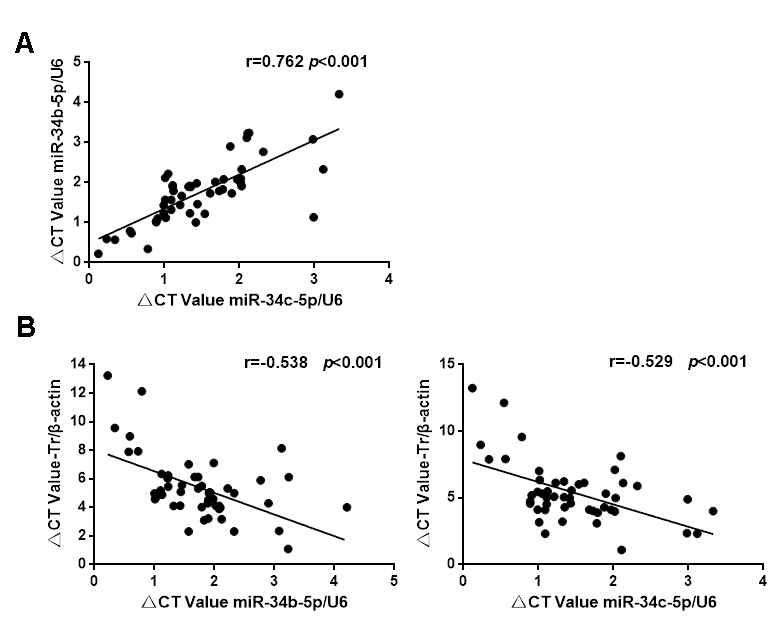

Supplement: Supplementary file 2 [file CPR-52-e12527-s002.tif]

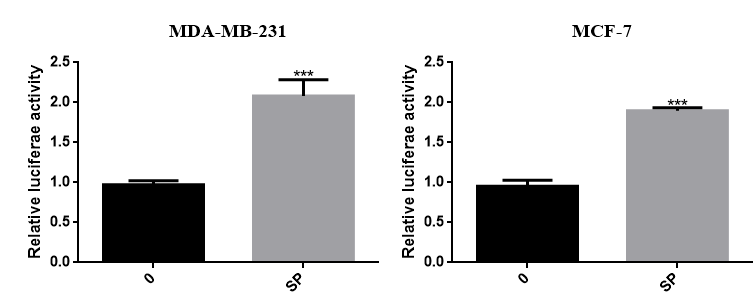

Supplement: Supplementary file 3 [file CPR-52-e12527-s003.tif]

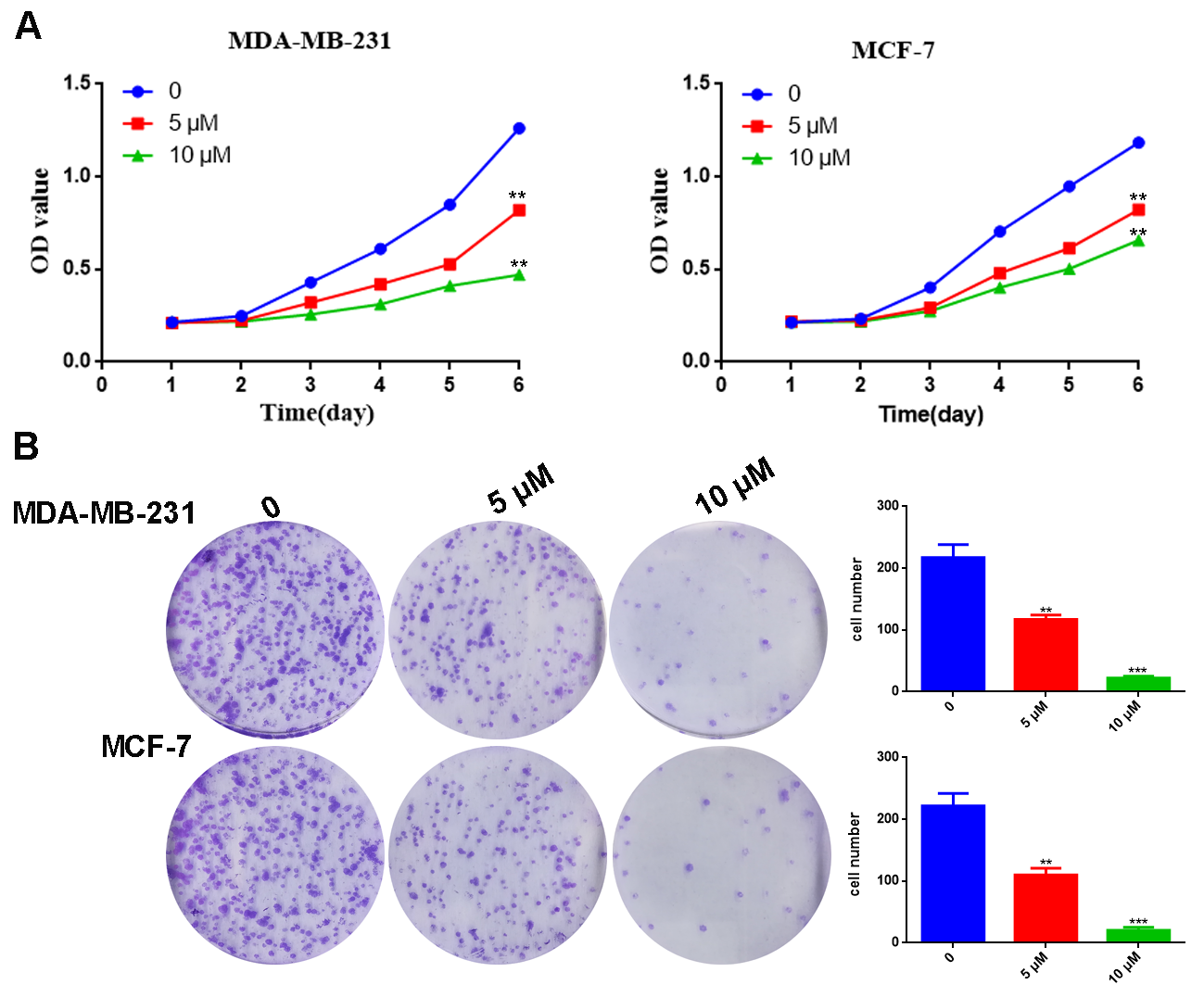

Supplement: Supplementary file 4 [file CPR-52-e12527-s004.tif]

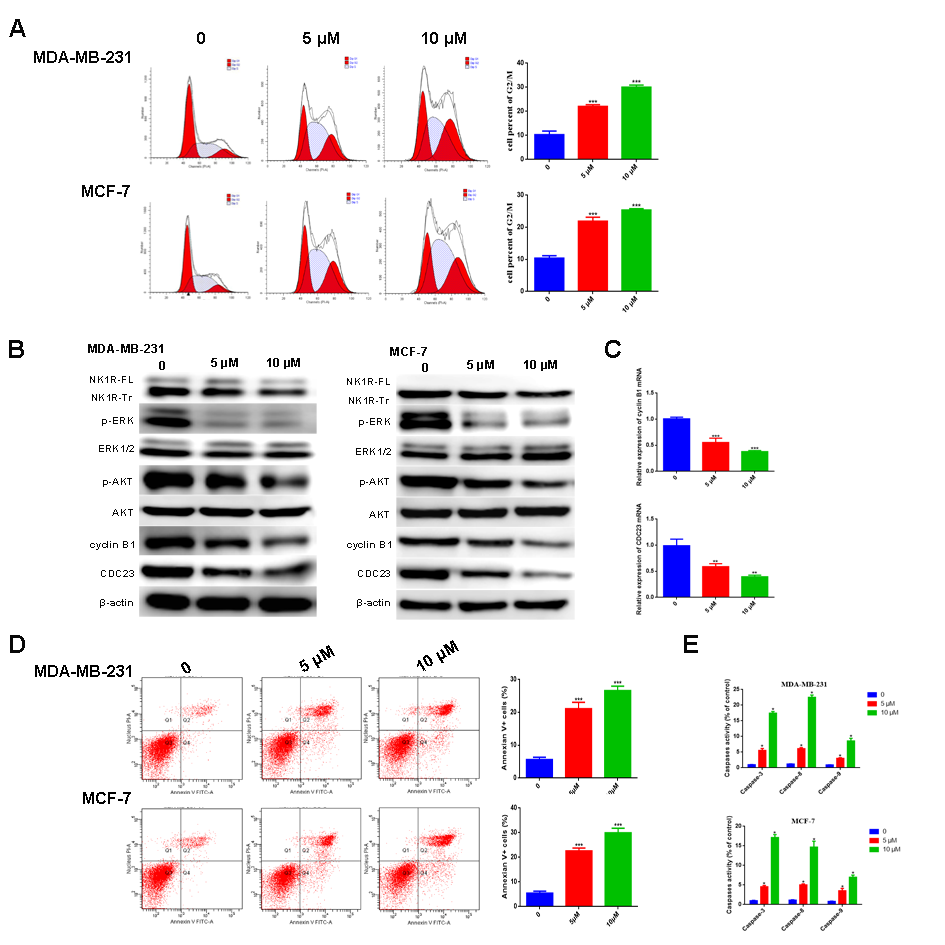

Supplement: Supplementary file 5 [file CPR-52-e12527-s005.tif]

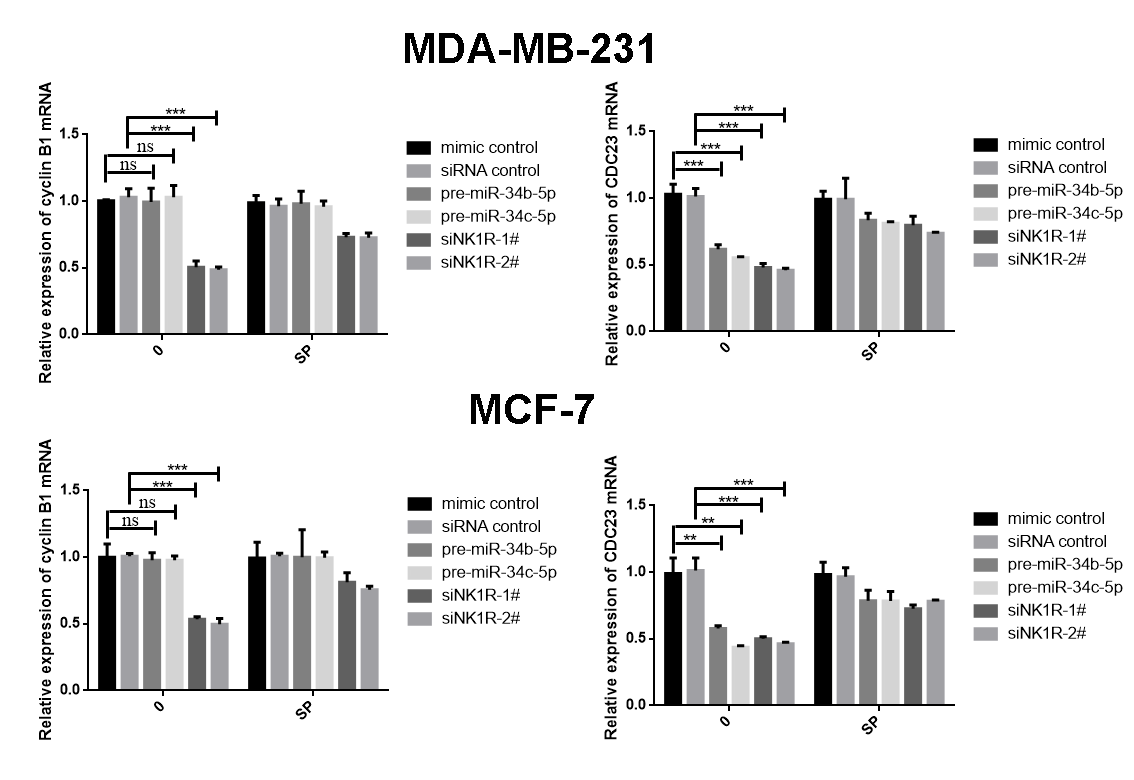

Supplement: Supplementary file 6 [file CPR-52-e12527-s006.tif]
