# Supplementary material for: MiR‐34b/c‐5p and the neurokinin‐1 receptor regulate breast cancer cell proliferation and apoptosis
Source: Cell Prolif. 2018 Oct 17;52(1):e12527. doi: 10.1111/cpr.12527 (PMC6430481; doi:10.1111/cpr.12527)
Supplement: Supplementary file 7 [file CPR-52-e12527-s007.docx]

**Supplementary table S1. Spearman correlation analysis between miR-34b/c-5p and NK1R expression and Clinicopathological parameters in breast cancer**

| Clinicopathological parameters | Expression of miR-34b-5p  Spearman correlation *p* | | Expression of miR-34c-5p  Spearman correlation *p* | | Expression of NK1R-Tr  Spearman correlation *p* | | | Expression of NK1R-FL  Spearman correlation *p* | | |
| --- | --- | --- | --- | --- | --- | --- | --- | --- | --- | --- |
| Age  TNM stage  Lymph node status  ER  PR  HER-2  Ki-67(%) | 0.209  -0.339  -0.220  -0.080  -0.038  -0.263  -0.530 | 0.144  0.016*  0.124  0.583  0.793  0.064  <0.001** | 0.173  -0.315  -0.098  -0.042  -0.160  -0.384  -0.436 | 0.228  0.026*  0.496  0.772  0.266  0.006**  0.002** | | -0.169  0.300  0.220  0.290  0.283  0.235  0.317 | 0.241  0.034*  0.125  0.041*  0.047*  0.101  0.025* | | 0.158  0.129  0.325  0.025  -0.138  -0.169  -0.043 | 0.273  0.372  0.021*  0.864  0.338  0.240  0.767 |

*, *p*<0.05; **, *p*<0.01.
