# Supplementary material for: MiR‐34b/c‐5p and the neurokinin‐1 receptor regulate breast cancer cell proliferation and apoptosis
Source: Cell Prolif. 2018 Oct 17;52(1):e12527. doi: 10.1111/cpr.12527 (PMC6430481; doi:10.1111/cpr.12527)
Supplement: Supplementary file 8 [file CPR-52-e12527-s008.docx]

**Supplementary table S2. Results of half inhibition (IC50) experiments in breast cancer cell lines following the administration aprepiatiant**

| Cancer cell line | IC_50_ (μM) |
| --- | --- |
| MDA-MB-231 | 19.2 |
| MCF-7 | 21.1 |
